# Lupus mastitis and antiphospholipid syndrome treated with anticoagulation and immunosuppression: a case report

**DOI:** 10.1186/s13256-023-04054-1

**Published:** 2023-08-09

**Authors:** Lauren J. He, Laarni C. Quimson, Oluwakemi Onajin, Kimberly C. Trotter

**Affiliations:** 1 Department of Medicine, University of Chicago Medical Center, 1000 E. 53rd St, Apt 412S, Chicago, IL 60615 USA; 2 Section of Rheumatology, University of Chicago, Chicago, IL USA; 3 Section of Dermatology, University of Chicago, Chicago, IL USA

**Keywords:** Lupus mastitis, Antiphospholipid syndrome, Breast lesion, Case report, Lupus erythematosus panniculitis

## Abstract

**Background:**

Systemic lupus erythematosus is an autoimmune disease that can have cutaneous and systemic manifestations. Lupus panniculitis, also known as lupus mastitis, is a subset of chronic cutaneous lupus erythematosus that involves inflammation of the subcutaneous fat. The pathogenesis of lupus mastitis is not fully understood. Diagnosis involves a combination of skin manifestations, imaging, and pathologic confirmation. Treatment typically includes steroids and antimalarials, with more severe disease requiring additional immunosuppressive medications. This report highlights a case of lupus mastitis treated with rituximab and a possible relationship between this disease process and thrombotic disease.

**Case presentation:**

A 48-year-old African American female with systemic lupus erythematosus and antiphospholipid syndrome presented with new breast lesion. Mammography revealed calcifications and increased density with coarse trabecular pattern. Breast biopsy showed features of cutaneous lupus and occlusive vasculopathy. The patient was diagnosed with lupus mastitis and treated with anticoagulation, rituximab, mycophenolate mofetil, and quinacrine with resolution of her symptoms.

**Conclusion:**

This patient experienced improvement in her breast symptoms with combination therapy including rituximab. There are only two other cases reported in literature of patients with lupus mastitis responding to rituximab, highlighting the possible role of B cell depleting therapy for those who have contraindications to standard treatments for lupus mastitis. While the pathophysiology of lupus mastitis is thought to be immune driven, some literature suggests that associated thrombosis commonly seen may be due to a physiologic overlap similar to antiphospholipid syndrome. The possible relationship between antiphospholipid syndrome and lupus mastitis and the use of antiplatelet and anticoagulation therapy is discussed and may warrant further investigation.

## Background

Lupus mastitis is a rare form of panniculitis occurring in patients with systemic or discoid lupus. Lupus panniculitis refers to inflammation of the subcutaneous fat and is termed lupus mastitis when affecting the breasts. There have been 36 cases of lupus mastitis described in literature thus far [1]. This disease occurs mostly in females with an average age of 40 years and can present as single or multiple subcutaneous or deep breast masses that can be tender and painful with erythema or violaceous discoloration. The clinical course is usually chronic with remission and flares and can present either at the same site or at different sites. Diagnosis requires a combination of imaging and biopsy, with major histopathologic criteria including hyaline fat necrosis, lymphocytic infiltration with lymphoid nodules surrounding necrosis, periseptal or lobular panniculitis, and microcalcifications [1]. We report a case of lupus mastitis complicated by antiphospholipid syndrome and explore the clinical overlap of these conditions and treatment options.

## Case presentation

**Fig. 1:**
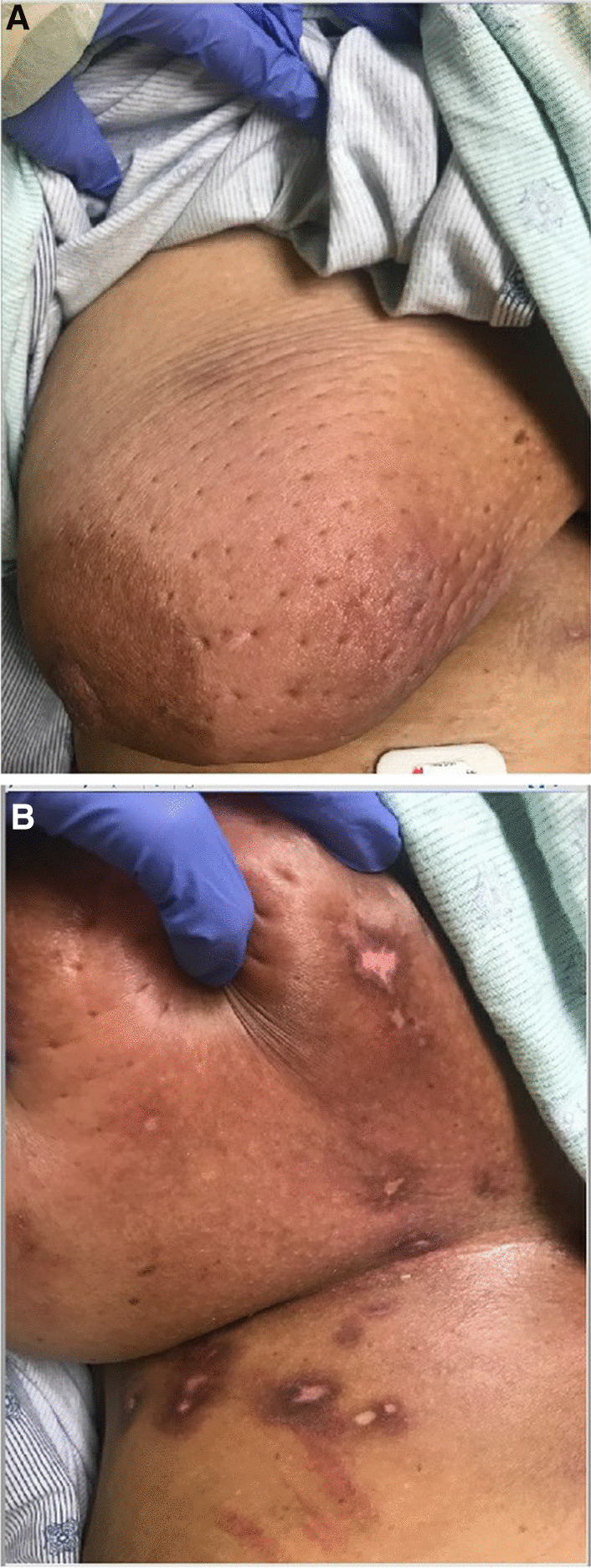
Right breast lesions medial (**a**) and inferior (**b**)

**Fig. 2:**
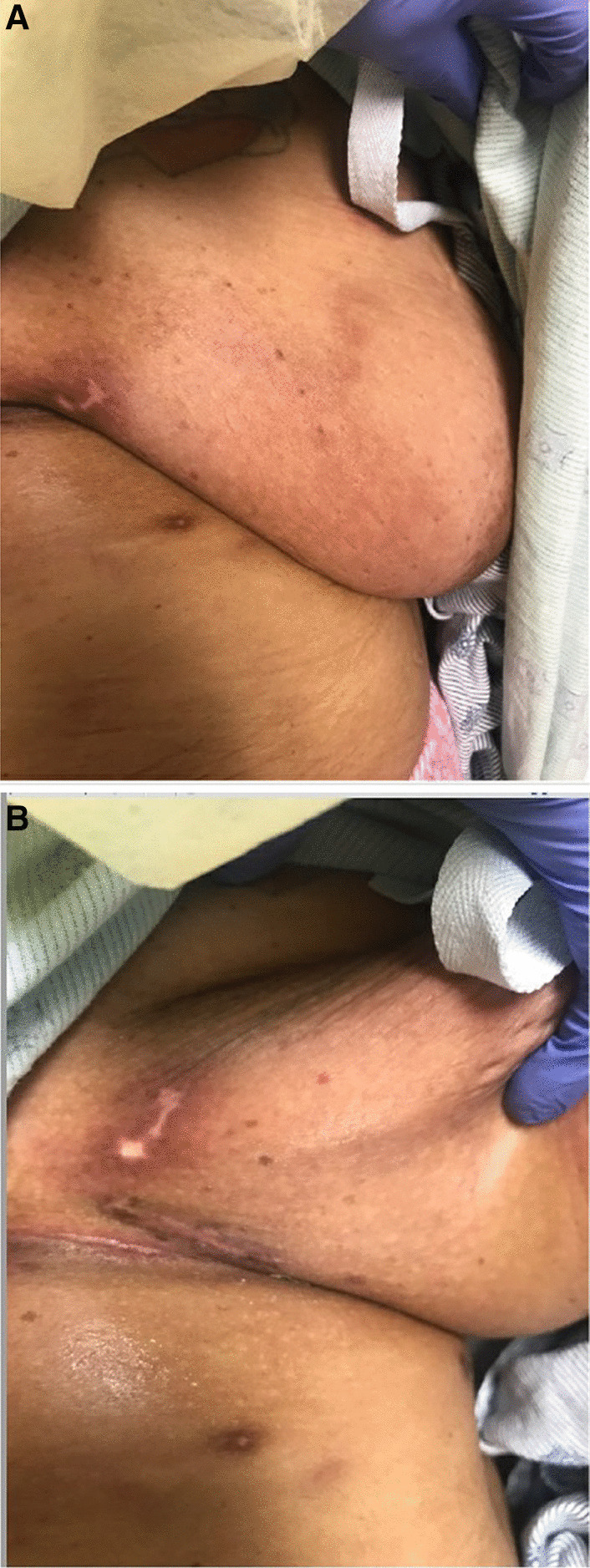
Left breast lesions medial (**a**) and inferior (**b**)

A African American woman in her forties with a history of systemic lupus erythematosus (SLE) and antiphospholipid syndrome (APS) was admitted to the hospital with scalp rash, alopecia, and breast lesions for 3 weeks (Figs. 1, 2). She was originally diagnosed with SLE in 1999, with alopecia, malar rash, inflammatory arthritis, arthralgias, scleritis, and laboratory data including positive ANA, ds-DNA, SSA, and hypocomplementemia. She also had concomitant antiphospholipid syndrome on the basis of bilateral partially occlusive deep vein thromboses (DVT) in the left innominate, subclavian, axillary, and brachial veins, as well as near-complete occlusion of the superior vena cava (SVC). Laboratory data supporting this diagnosis included persistently detectable anti-cardiolipin IgM antibody and the presence of lupus anticoagulant using aPTT-based system: PTTLA screen prolonged at 52.7 seconds (ref < 42.6 seconds), PTTLA mixing phase prolonged at 42.4 seconds (ref < 38.0 seconds), and confirmatory phase clotting time corrected by 32.8% (ref < 11.3%) in the presence of high concentration phospholipid.

Medications at the time of presentation included prednisone 10 mg daily, mycophenolate mofetil 1500 mg twice a day, and quinacrine 100 mg daily. She received one dose of rituximab 1000 mg 4 weeks prior during an inpatient admission due to severe, refractory cutaneous disease and arthritis with partial improvement in her symptoms. She was not on hydroxychloroquine due to retinal toxicity and had previously failed azathioprine and methotrexate due to persistent SLE flares. Belimumab was considered, but we opted to give rituximab given the severity of her disease. Given her history of T cell lymphoma and stem cell transplant, as well as lack of renal or other internal organ involvement, cyclophosphamide was not initiated.

Physical examination revealed multiple stellate, atrophic white patches and plaques with surrounding erythema and hyperpigmentation on the scalp and bilateral breast and erythema, warmth, and induration of the medial breasts. Laboratory results were significant for C3 of 45 mg/dL, C4 13 mg/dL, anti-dsDNA 21.2 [iU]/dL, CRP 11 mg/L. CBC showed a mild leukopenia of 3.0 × 10^3^/μL. CMP was normal. APS testing was notable for anti-cardiolipin M Ab 30.7 MPL unit and B2-glycoprotein Ab IgG 35.0.

**Fig. 3:**
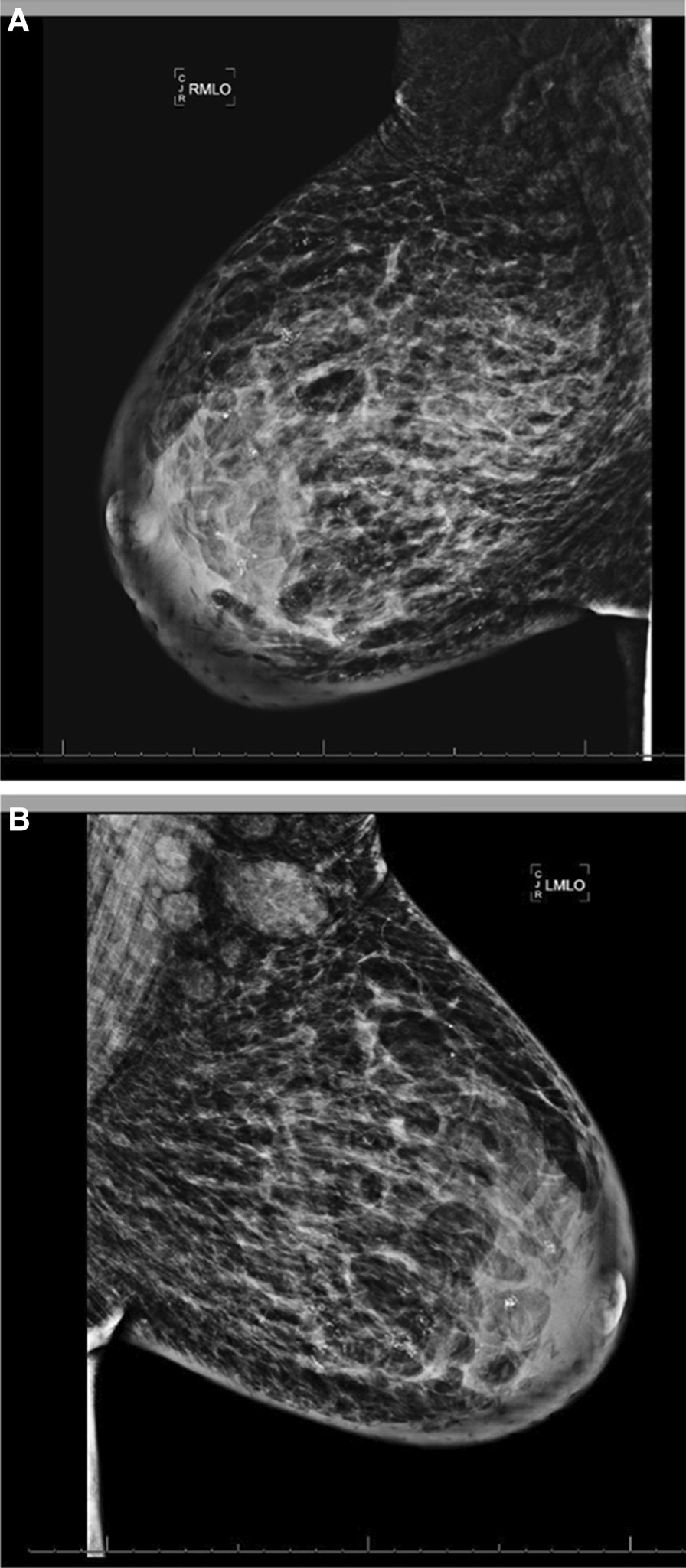
Mammogram of bilateral breasts (**a** right breast,** b** left breast) with skin thickening, calcifications, and heterogeneous increased density with a coarse trabecular pattern

**Fig. 4:**
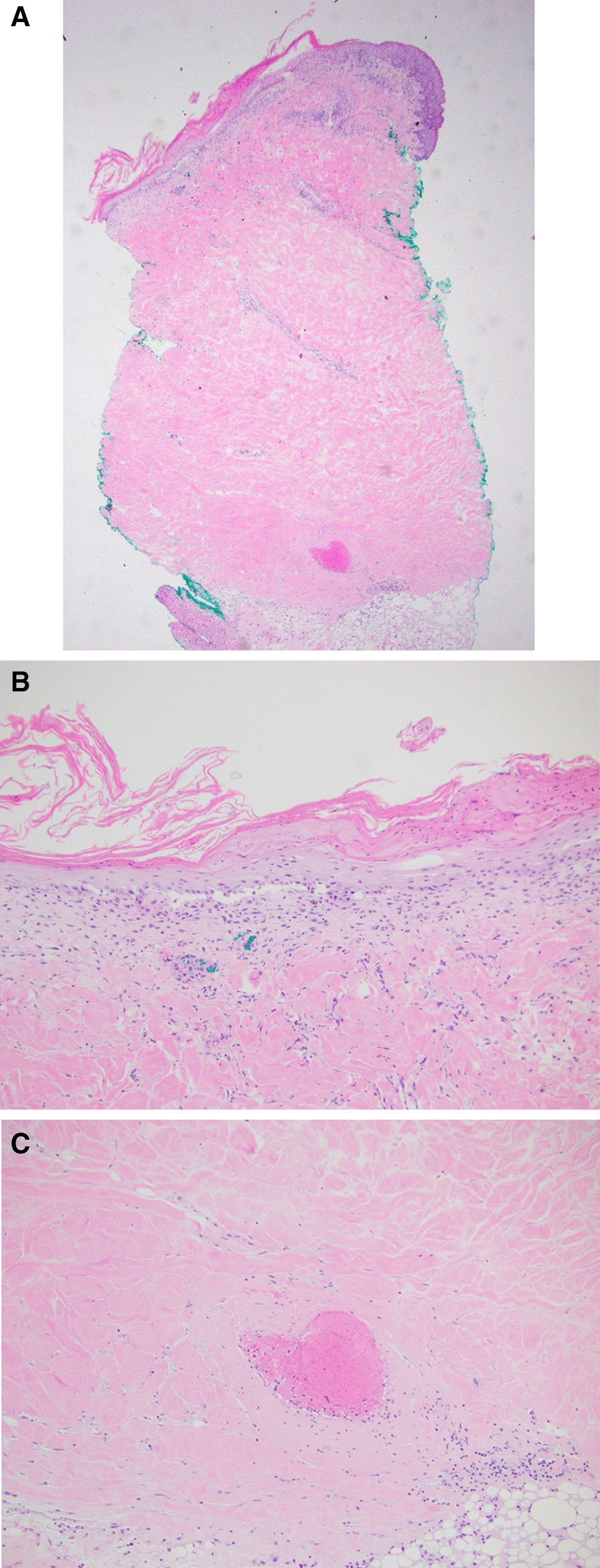
Skin biopsy demonstrating vacuolar interface dermatitis, ischemic dermis, and increased mucin with lymphocytic infiltration of the vessel wall (**a**, **b**). Occlusive thrombus and fibrinoid necrosis is demonstrated in the deep dermis (**c**)

The breast lesions did not improve despite fluconazole, nystatin powder, oral doxycycline, and IV clindamycin. Mammogram revealed skin thickening, calcifications, and increased density with a coarse trabecular pattern concerning for lupus mastitis (Fig. 3). Computed tomography (CT) scan showed moderately enlarged bilateral axillary lymph nodes, asymmetric skin thickening, and fatty stranding in the bilateral breast without discrete  abscess. CT scan also demonstrated near-complete occlusion of the superior vena cava and left brachiocephalic vein with extensive venous collateralization. Lymph node biopsy was negative for malignancy. A skin biopsy of a breast lesion demonstrated hyperkeratotic and atrophic epidermis with vacuolar interface dermatitis, wedge-shaped area of ischemic dermis, and increased mucin (Fig. 4A, B). Lymphocytic infiltration of the vessel wall with occlusive thrombus and fibrinoid necrosis was also identified in the deep dermis (Fig. 4C). These histopathologic features were compatible with her history of APS and SLE.

The patient was diagnosed with lupus mastitis. She was treated with prednisone 40 mg (with taper) and continued on mycophenolate mofetil (MMF). She received her second dose of rituximab 1000 mg during this hospitalization. The decision to continue rituximab was based on her initial improvement on this regimen. She was additionally started on enoxaprin for APS. At her 6-month follow-up appointment, her breast symptoms had resolved. The decision was made to continue MMF, quinacrine, rituximab every 6 months, prednisone 10 mg, and enoxaparin. One year after diagnosis, her symptoms continued to be well controlled on this regimen.

## Discussion and conclusion

Lupus mastitis is a rare form of lupus panniculitis that usually occurs in patients with longstanding disease, although it has also been reported as an initial disease manifestation [1, 2]. The most common presentation is tender subcutaneous breast nodule(s) with erythema or violaceous discoloration, however manifestations can range from asymptomatic disease to more severe features including nipple involvement, discharge, ulceration, or lymphadenopathy.

Radiographic features include breast density associated with calcifications due to fat necrosis, consistent with our patient’s imaging findings. As surgical intervention and tissue-damaging biopsies risk worsening the disease, specific clinical and radiographic classifications are the primary focus for diagnosis. When biopsies are pursued, single attempts and fine needle aspiration (FNA) are favored to avoid exacerbating underlying disease. Histologically, hyaline fat necrosis is the hallmark finding in lupus mastitis [3]. It is also associated with dense lymphoid infiltrates (usually in a lobular distribution), fibrosis, and calcifications. An additional common pathologic feature is lymphocytic vasculitis affecting medium and small vessels.

For mild disease, treatment includes topical glucocorticoids and antimalarials [4]. More severe forms are treated with aggressive immunosuppression including high-dose oral corticosteroids, azathioprine, methotrexate, cyclophosphamide, and cyclosporin. Our patient had progressive disease despite high-dose steroids, azathioprine, mycophenolate mofetil, methotrexate, and quinacrine and was unable to use hydroxychloroquine owing to retinal toxicity. Other traditional therapies were deferred given her history of T cell lymphoma with stem cell transplant and multiple inpatient admissions. Thalidomide has also been studied, although its use is limited by the frequent occurrence of neuropathy [5]. Interestingly, our patient experienced resolution of symptoms after two doses of rituximab. There have been two other cases of refractory disease that responded to rituximab, indicating a possible role for B-cell depleting therapy in some circumstances [6, 7].

Anticoagulation is not commonly used in treatment of lupus mastitis. However, there have been reported cases of antiphospholipid-associated panniculitis in SLE for which antiplatelet therapy has been used [8, 9]. Hunt *et al.* [8] reported a 60-year-old man who presented with skin nodules affecting the legs, arthralgias, and elevated anti-beta-2 glycoprotein IgM and anticardiolipin IgG. Biopsy revealed septal fibrosis and fat necrosis with perivascular lymphocytic infiltrate suggestive of panniculitis. Symptoms improved with low-dose aspirin. Other case reports of patients with positive antiphospholipid serology and panniculitis report clinical response to prednisone and antiplatelet therapy [9–11]. These patients had no other thrombotic events. There have been few case reports published of patients with APS and lupus panniculitis who were treated with immunosuppression and anticoagulation with minimal clinical response [12].

Our patient’s histologic findings of occlusive vasculopathy were more consistent with her known antiphospholipid syndrome. As this was a more superficial skin biopsy, a deeper incision skin biopsy may have been helpful to better characterize histologic findings. Our patient’s clinical features of established SLE, breast pain and rash with axillary lymphadenopathy, mammographic findings of increased breast density and calcifications were consistent with the diagnosis of lupus mastitis. She had a remote history of deep vein thrombosis (DVT) and superior vena cava thrombus but had previously deferred anticoagulation therapy. Given the histologic findings of occlusive thrombosis and prior clots, anticoagulation with enoxaparin was started. The underlying pathophysiology of panniculitis in patients with SLE is thought to be mostly driven by an autoimmune physiology, as histopathology typically reveals immune complexes at the dermal–epidermal junction and blood vessels in affected areas [13]. However, there is ongoing consideration that thrombosis in the veins of the subcutaneous fat may be more directly associated with APS in patients with known disease [9]. This distinction is important as it could change the treatment course for patients with positive antiphospholipid serology without previous thromboembolic events who develop panniculitis. This case highlights the importance of further exploring the relationship between APS and lupus panniculitis and the role of anticoagulation therapy, and adds to the existing literature on the possibility of treating patients with lupus mastitis with rituximab when other therapies are limited.

## Data Availability

All patient data and materials are available upon further request.
